# Anticipating Moral and Economic Considerations, Opportunities, and Potential Frictions for AI in Medical Imaging: Multistakeholder Cocreation Study

**DOI:** 10.2196/83407

**Published:** 2026-02-25

**Authors:** Martin Bastiaan Schilder, Alexandra Keyser, Susan van Hees, Alessandro Sbrizzi, Wouter Pieter Christiaan Boon

**Affiliations:** 1 Computational Imaging Group for MR Therapy and Diagnostics, Center for Image Sciences, University Medical Center Utrecht, Heidelberglaan 100, Utrecht, 3508GA, The Netherlands, 31 887569270; 2 Faculty of Geosciences, Copernicus Institute of Sustainable Development, Utrecht University, Utrecht, The Netherlands

**Keywords:** cocreation, stakeholder perspectives, radiology, artificial intelligence, responsible research and innovation

## Abstract

**Background:**

Artificial intelligence (AI) promises to significantly impact daily radiology practices. Numerous studies have already been conducted that anticipate this potentially disruptive innovation. So far, these studies have mainly focused on single topics, such as “trust,” or investigating perspectives of single stakeholder groups, such as “radiologists.”

**Objective:**

This study aims to explore future directions for AI in radiology by incorporating perspectives of a heterogeneous group of stakeholders on a broad spectrum of moral and economic topics. It also aims to cocreate and reflect with a broad range of stakeholders on viable implementation scenarios for scalable AI applications in radiology in the Netherlands, thereby identifying potential opportunities and frictions, with a focus on moral and economic considerations.

**Methods:**

To inform the workshop design, a nonsystematic narrative literature search was performed to deepen our understanding of key moral and economic considerations at play in the field of radiology and AI. Workshop participants, representing a wide range of actors including radiologists, innovators, and patient representatives, were selected using purposive sampling. Data were collected in a cocreation workshop. In 3 subsequent rounds, mixed over 3 breakout groups, a total of 17 participants were asked to (1) map what they considered important moral and economic considerations, (2) envision possible future scenarios for AI in radiology, and (3) discuss opportunities, frictions, and routes to success. Transcribed recordings were coded and cross-checked.

**Results:**

Workshop participants envision future AI-driven scenarios, ranging from extramural imaging departments for increased accessibility to health care, to multimodal data integration for human-centered AI-enhanced diagnostics. Seven themes emerge from the discussions during the workshop: (1) trust and efficiency of AI technologies, (2) responsibilities in clinical decision-making when AI is involved, (3) diagnosis as a one-off versus an iterative process, (4) regulations as a requirement or a restriction, (5) economic benefits or drawbacks, (6) trade-off between amount of information required and patient privacy, and (7) environmental considerations.

**Conclusions:**

Reflecting on the 7 emerging themes, we identify three overarching topics: (1) human-AI collaboration and trust, (2) governance, regulation, and ethical safeguards, and (3) value creation and sustainability. These topics highlight the need to balance technological advancements with ethical responsibility, institutional accountability, and societal benefit. They also underscore the importance of designing AI systems that not only perform well but are also trusted and aligned with clinical workflows and patient values. These overarching themes offer a lens through which future research and policy can navigate the complex interplay between innovation, regulation, and real-world implementation. Future research is needed to validate the generalizability of the results across various countries and health care settings.

## Introduction

### Background

As health care systems worldwide face increasing demand and complexity, the integration of advanced technologies such as artificial intelligence (AI) is gaining momentum. Many clinical experts and sector specialists consider AI to have transformative potential for the field of radiology [1-4]. They argue, for instance, that AI offers the potential to enhance diagnostic accuracy, to support early disease detection, and to personalize patient care through applications such as image segmentation, anomaly detection, and predictive modeling [1,5-7]. These applications could automate routine tasks and uncover subtle patterns that can be missed by the human eye [1,8] and help radiologists to focus more on complex clinical decisions such as diagnosing intricate disease presentations [7].

There is a growing body of literature discussing the promises of AI in radiology from different perspectives. From a moral perspective, many scholars propose AI to contribute to safer, more patient-centered care by reducing diagnostic errors and unnecessary interventions [9]. Economically, proponents argue that AI can contribute to streamlining radiology workflows by improving productivity, shortening turnaround times, and lowering health care costs through early detection of disease and more efficient resource allocation [7,10-12]. Alongside these promises, scholars warn that we should remain sensitive to risks and challenges, such as algorithm opacity and profit-driven motives, created by an emerging role of AI in health care [13,14].

Despite these promises, much of the existing research on the role of AI in radiology remains limited in scope. So far, various studies have explored AI in radiology, mostly from a single perspective, such as trust or acceptance [15,16]. Moreover, existing research has primarily explored these perspectives within only a single stakeholder group or a limited range of groups, such as radiologists, innovators, or patients [7,15,17-20]. These studies also have predominantly applied retrospective methods, such as surveys and questionnaires [16,20-22]. Although these earlier studies offer valuable insights, there is a need for research broadening the perspectives and stakeholders. Adopting a broader approach enables the examination of interactions among stakeholders while situating their perspectives within the wider set of trade-offs and contextual factors.

### Objective

The study aimed to cocreate and reflect with a broad range of stakeholders on viable implementation scenarios for scalable AI applications in radiology in the Netherlands, thereby identifying potential opportunities and frictions, with a focus on moral and economic considerations. Drawing on insights from responsible innovation, we endeavored to include a wide variety of views in an early stage of the emergence of a technology, anticipating potential implications and frictions, aiming to increase chances for successful innovations [17,23,24]. The inclusion of varied perspectives, especially those of patients [25,26], has been widely advocated as essential to shaping relevant research agendas and outcomes [15,16,27,28]. Early-stage engagement should increase opportunities for aligning technology development not only with clinical needs and regulatory requirements but also to include current experiences and care practices, anticipate expectations and underlying values [17,27,29-33], and answer questions regarding commercialization [34]. Engaging stakeholders encourages iterative feedback, increasing the chance of AI systems to evolve alongside real-world needs and cultivate shared ownership of the innovation process [8].

During a cocreation workshop, stakeholders were asked to build concrete implementation scenarios for AI in imagined future intra- and extramural settings and to reflect on expected opportunities and potential frictions created by moral and economic considerations. The forward-looking, participatory approach that we used contributed to a more comprehensive understanding of possible future pathways, which we present in the “Results” section. Subsequently, we describe the 7 themes that emerged from these scenarios, touching on various moral and economic considerations, involving among others the role of the human (radiologist and patients), as well as issues related to regulations, economic considerations, and the environmental impact. Finally, the “Discussion” section further reflects on these themes, subsequently concluding with 3 key implications for futures of AI in radiology.

## Methods

### Study Design

Our research approach reflects starting points from responsible innovation. We started with a nonsystematic narrative literature search to identify existing topics and gaps in the scientific discourse. These topics served as input for the cocreation workshop in which future scenarios for AI in radiology were developed. In the workshop, we took an explorative, anticipatory, and cocreation approach, in which stakeholders were allowed to raise and reflect on interconnected topics, rather than addressing these topics in isolation.

### Identification of Workshop Topics

To gain an initial understanding and to provide the research team with context of the recurring topics and stakeholders in the field of AI, radiology, and business models, we conducted a nonsystematic narrative literature search aimed at identifying key topics and trends in the field [35,36]. In light of the fact that we aimed for a broad group of topics and stakeholders, we were a priori interested in both moral and economic considerations. Resultingly, our approach included keyword-based searches in primarily PubMed and Google Scholar using search terms such as “Radiology,” “AI,” “Transparency,” and “Value proposition.” These terms were iteratively refined as understanding of the topic evolved and, in the end, also included “Algorithmic fairness” and “Revenue streams” as search terms, as is common practice in a narrative review [36]. We also used forward and backward snowballing from key publications to capture additional influential works. In addition, we browsed the digital archives of leading journals in the field, including *Journal of Medical Internet Research* (and relevant sister journals), *European Radiology*, and *Insights into Imaging*. This flexible and exploratory search strategy suited our objective of mapping conceptual developments and identifying recurrent topics across the literature rather than providing an exhaustive or systematic synthesis. Textbox 1 provides the main findings of this review.

Textbox 1. **Topics covering moral and economic considerations coming from the literature search.**

In the literature search, we focused on artificial intelligence (AI), radiology, and business models, and both moral and economic considerations came forward. Radiologists increasingly rely on digital tools, including AI-based tools, for various functions [5-7]. This raises moral and economic concerns related to discussions about, for example, trust between the patient and physician [19,31,37-39], supervision and accountability in clinical decision-making [16,40,41], lacking or unclear reimbursement structures [42], and high upfront costs [43]. In these discussions, some recurring topics came forward.

Trust is often mentioned in literature as playing a critical role in the field of radiology. Where, traditionally, trust between a patient and a physician was deemed important, another dimension is added with the introduction of AI, and the definition of trust is increasingly broadened to both the clinicians’ and patients’ trust in the technology’s performance and reliability [15,18,33,44-49]. However, trust is a complex concept that relates to interconnected discussions on, among others, fairness, safety, explainability, and transparency [50]. To increase fairness and safeguard vulnerable populations, managing risks is essential [40,51]. AI models often reflect biases inherent to insufficiently diverse training datasets [40,51,52]. Resultingly, model performance can deteriorate if applied to a different population. Explainability and transparency are fundamental to building trust in AI systems [16,53,54] and can be considered key fairness strategies to mitigate bias and improve accountability [30]. Here, the distinction is that interpretability means that a radiologist can understand and make sense of the outputs of the AI system, whereas explainability means that the AI system also provides the radiologist with a rationale or reasoning behind the results [2].

Despite their importance, many AI models suffer from the “black box problem,” where decision-making processes can be opaque even to developers [30,46]. Obstruction of error identification through a lack of transparency compromises trust and accountability [30,55,56]. The General Data Protection Regulation mandates transparency and explainability in automated decision-making, but many systems struggle to meet this standard [46,57]. On the topic of data, while the General Data Protection Regulation, for instance, grants patients ownership over personal data through, for example, obliging data users to obtain informed consent, uncertainties persist regarding data reuse and consent withdrawal [40,46,55,58]. Ownership thus means that a person or legal entity has the full and inherent right to decide what happens with the data without requiring consent from anyone else. By contrast, controls refer to the delegated ability to use or manage data within boundaries agreed upon by the owner, granted through informed consent. Developers, users, and health care providers must navigate complex questions about responsible data storage and use [49]. Transparency in data management could further support trust, yet proprietary algorithms and cross-disciplinary misalignments between developers and health care practitioners create significant challenges [58]. It has therefore been advocated that radiologists must develop new skills to effectively collaborate with AI, which requires additional training [49,50,59,60] and potentially a shift in roles and responsibilities [61]. Concerns about overreliance on AI systems, loss of diagnostic expertise, and shifts in job satisfaction are frequently noted [62-64]. Additionally, unresolved issues in assigning accountability for AI-assisted errors complicate the integration into clinical workflows, raising questions about liability and autonomy among developers, users, and insurers [16,40,41].

Business models for AI applications in radiology outlined in the literature reviewed include perpetual licensing, pay-per-use, and subscription-based models [65,66], with most companies offering subscription or hybrid pricing models [65]. Deployment and pricing strategies have not yet converged to a preferred or market-dominant standard, and most vendors offer multiple options. Literature discusses the shift of software as a medical device from product-centric to platform-based models [67]. This idea of platformization could potentially facilitate easier (vendor-neutral) compatibility and integrability within existing hospital infrastructure [8,68,69]. In essence, all businesses in the medical software industry operate in a highly dynamic environment with constant challenges, including disruptive innovation, strict regulations, sustainability concerns, and uncertainties [70,71]. To navigate this landscape and mitigate risk, a more dynamic business model has been proposed, tailored to the specific demands of the software as medical device domain. Long-term stability of business models can be achieved through diversified revenue streams and optimizing operational efficiency, regardless of market conditions [71]. Hospitals also seek ways to fund AI-supported services. Some sources point to missing reimbursement structures or high upfront costs, hindering adoption [42,43]. There is, however, a need for more research on sustainable business models for hospitals that balance adoption costs with strong value propositions and reimbursement frameworks [72,73] and value assessment through real-world clinical validation [74].

The insights from this nonsystematic narrative literature search were synthesized into topics, prompts, and discussion points, which served as input for the cocreation workshop, and they were used to create workshop canvasses. The workshop canvasses can be found in MultimediaAppendices 1  2. These canvases guided participant engagement and helped ensure that the workshop addressed research-relevant issues by suggesting conversation topics, while remaining flexible for participant input.

### Workshop Design and Participants

Since it is argued that integration of AI into radiology may provide a transformative opportunity for health care delivery, we further explored opportunities for AI in radiology in a multistakeholder workshop. Workshop participants were selected using purposive sampling to ensure the inclusion of individuals relevant to the research objectives. We aimed to include participants from diverse stakeholder backgrounds. As a point of departure, participants from an earlier workshop with a diverse panel of stakeholder backgrounds [28,61] were approached first. In addition, we explicitly looked for expertise and relevant types of stakeholders that were missing, were underrepresented, and regarded as important in relation to the workshop’s themes. This search included reaching out to contacts in the authors’ own network, for example, through LinkedIn and institutional websites. Prospective participants were also allowed to suggest relevant candidates from their own network. The patient representatives were members of the hospital patient council and were approached by hospital staff for participation. Participation was voluntary aside from a compensation for travel-related expenses. Potential selection bias was balanced by having participants representing very different perspectives.

In the workshop, patient representatives (n=2), radiologists (n=2), laboratory technicians (n=2), a technology developer (n=1), clinical researchers (n=2), industry employees (imaging hardware company employee: n=2; medical software company employee: n=1), a funding body employee (n=1), a clinical physicist (n=1), a business developer (n=1), and social scientists (n=2) participated, totaling 17 participants. The sessions were moderated by the researchers (MBS, SH, and WB). Next to discussing from their expert point of view, all participants were also invited to reflect on questions from a general citizen’s point of view, allowing for flexible conversations between the participants.

### Ethical Considerations

The research protocol was assessed and exempted from medical ethical review by the Medical Ethics Board of University Medical Center Utrecht (Declaration METC no. 22‐475/DB, d.d. 1 March 2022) [75]. This exemption was determined in accordance with the Dutch Medical Research Involving Human Subjects Act and the definitions and guidance provided by the Central Committee on Research Involving Human Subjects [76]. All participants gave written and verbal informed consent prior to the workshop and had the opportunity to react to a written workshop report after the workshop. In the analysis, the data were coded, meaning that participants received a study number. In this paper, we refer to the participants with that same code, although sometimes referring to their respective stakeholder background. Patient representatives received compensation for travel-related expenses and were offered a voucher of €25 (approximately US $27 at the time of the workshop).

### Data Collection

The cocreation workshop took place in June 2024 and lasted 3 hours. Participants were asked in three rounds to (1) map and reflect on moral and economic considerations they considered important when thinking about the field of radiology and AI, (2) cocreate concrete scenarios, envisioning plausible futures for radiology with AI in the not too long future (year 2040), to elaborate on the broader implications of their considerations to the field, and (3) think about opportunities and potential frictions related to these scenarios and how to deal with or prevent them.

The 15-year time frame was chosen to allow participants to envision a future where significant transformation could happen, while still being close enough to make the ideas feel actionable. We asked our participants to envision scenarios *postadoption* of AI in radiology, intentionally probing the workshop participants not to focus on pragmatic topics, such as hardware requirements, because our aim was to explore strategic and value-driven perspectives rather than operational constraints. We wanted participants to think beyond immediate technical hurdles and envision how AI could reshape workflows, roles, and patient care once adoption challenges have been resolved. In 3 breakout groups, each with 5 or 6 participants, scenarios were cocreated and discussed. In round 1, the participants were asked to discuss their respective considerations in groups of 2 or 3 participants. For rounds 2 and 3, we provided the participants with a worksheet canvas to guide the discussion. The questions on the canvasses were loosely based on the topics we found in our literature search (Textbox 1). The canvasses can be found in MultimediaAppendices 1  2. We explicitly told participants that these sheets could be used as inspiration for conversations, and if other ideas came to mind, that they were free to explore them. No further prompts were provided, except for those related to time management of the workshop. If questions on interpretation of the canvasses came forward, the session moderators answered the question neutrally and aimed not to steer the discussion in a certain direction. In case of dissent between the participants, the session moderators did not intervene, as disagreements between the participants could potentially reveal interesting frictions between various stakeholder backgrounds. All discussions were audio recorded, the filled-out worksheet canvasses were stored, and notes were taken by the researchers (MBS, SH, and WB). A more elaborate quotebook is provided in Multimedia Appendix 3.

### Data Analysis

The workshop was held in Dutch. All audio data were automatically transcribed using Amberscript (Amberscript BV), reread, checked, and corrected where necessary. Transcripts were then automatically translated to English using Microsoft Word (Microsoft Corporation), again reread, checked, and corrected where necessary. The transcripts were independently coded. MBS and SH, as native Dutch speakers, coded the original transcripts in order to stay close to the original meaning, and AK, as a native English speaker, coded the translated transcripts. The codes were subsequently compared and refined using qualitative analysis software NVivo (version 14; Lumivero). Data were analyzed using a semi-inductive coding approach, guided by predefined categories, but remaining flexible to the identification of emergent codes as new themes arose from the data. The initial codes were related to the topics we identified in the literature search and that we described in Textbox 1, namely, function, performance, supervision or autonomy, responsibility, explainability, transparency, training and job roles, algorithm performance, user and patient security, data management, accountability, bias, value assessment, business model aspects, value proposition, funding, platformization, and compatibility and integrability. Additionally, during the analysis, we found that “patient communication” was emerging as a new and recurring topic. This code was subsequently added to the codebook. Our analysis focused on moral and economic considerations, opportunities, and frictions, which materialized in the scenarios. To conform with scientific rigor, consistency between the researchers was ensured, as the researchers discussed their findings after initially independently coding the transcripts. That is, these peer debriefing sessions aided in ensuring that our interpretations were firmly grounded in the data. During these peer debriefing sessions, the researchers systematically compared their coding decisions for each transcript, focusing on whether the chosen codes accurately captured the meaning of the data and whether the interpretations were consistent across researchers. They reviewed the rationale behind each assigned code, discussed eventual discrepancies, and clarified ambiguous segments. Disagreements were openly discussed, and alternative perspectives were considered before reaching a shared interpretation, ensuring that the final coding reflected consensus. This iterative dialogue served to refine the coding framework and enhance the credibility of the analysis. Furthermore, a workshop report inspired by a preliminary analysis of the data was shared with the participants, providing them with an opportunity to disagree with the researchers’ interpretation of the data or add thoughts that came after the workshop.

## Results

### Cocreation Workshop

In this section, we share the findings, drawing on insights from the multistakeholder cocreation workshop. Many topics came forward, including questions regarding sharing or balancing responsibilities between humans and technology, power and influence in relation to data, ownership, access and trust, and questions regarding costs and payments. Developing and reflecting on the scenarios helped identify expectations. Different stakeholders brought forward different perspectives in the discussions. For example, in groups that included a patient representative, the importance of having a human radiologist involved came forward.

Most participants shared the expectation that between now and 2040 much will change and probably quite rapidly, referring to fast developments surrounding general purpose AI tools such as ChatGPT. Participants in our multistakeholder workshop considered in their scenario either intramural improvement of radiology processes or the extramural screening and early detection of disease. This resulted in 3 scenarios sketched out in the next paragraphs: AI copilot, scanner on tour, and Alzheimer disease (AD) screening. We present each scenario following a similar structure; first, we report what participants believe could be a good use case of AI (*materialization*), after which we share their *moral and economic considerations*, *opportunities,* and *frictions*. As a way of analysis, we highlight emerging themes from the scenarios.

### The 3 Scenarios

#### Scenario 1. Partner in Diagnosis: Envisioning AI as the Radiologist’s Trusted Copilot

##### Materialization

Participants envisioned that in 2040 AI would be integrated into every hospital’s radiology department, acting as a “copilot” to assist radiologists. Radiologists may grow from an executing role to a more supervisory role, sometimes required only to approve the AI’s suggestion. In this scenario, AI can aid in the noninvasive characterization of a tumor, enriching radiological findings on computed tomography or magnetic resonance imaging (MRI) with a description of histology and tumor biology without requiring surgical procedures, such as biopsies. Or, in case a rich collection of patient data is already available, AI can integrate such multimodal data:

...also the integration with biomarkers, with pathology with those [foundational models], and the EHR [Electronic Health Record], gives a much more complete picture than just with those images.” the workshop participant continued, arguing how that despite all clinicians already work together in the same system, she expects AI to be an enabler of actual integration : “(...) I think that integration into AI of all those different things will also become very strong.

[g3, s6]

AI in this use case supports the radiologist with additional insights that can be used for improved diagnosis or treatment response monitoring. According to the participants, the added value can be demonstrated in independent clinical studies in which researchers, clinicians, and ethical evaluation boards should have their usual role. Explicating added value incentivizes medical professionals to include AI in guidelines and insurance companies to reimburse its use. AI can subsequently be used to collect relevant patient data and provide comprehensive interpretations, whereas the radiologist retains ultimate responsibility for diagnosis and treatment decisions.

Participants thought that software developers should be in the lead in setting up a quality management system. They can supply their product, for example, through a pay-per-view business model, which is integrated into Picture Archiving and Communication System or electronic health record along with other vendors’ products. Users were regarded as being in the lead for evaluating real-world, postmarket applications. Although data have the potential to be part of business models, the participants thought that patients should retain ownership of their data, emphasizing that these data should be anonymous and nontraceable.

##### Moral and Economic Considerations

Participants deemed AI in radiology necessary to support the radiologist and underlined its potential, considering the hours spent on, for example, follow-up MRI scans without visible changes to the imaged pathology. Requirements formulated for this scenario were as follows: (1) The treating physician always remains responsible for the patient’s health. Since human experts should always remain in control, physicians remain responsible for communicating with patients. (2) AI should benefit the patients and lead to improved health, while applying to as many patients as possible. (3) Trust in AI can be increased by observing well-performing models that provide added diagnostic value, which can convince radiologists to use and fully adopt these models. Trust in AI was considered a means of adoption for this technology. (4) The industry should focus on user-friendliness and insightful cost-benefit analyses to improve adoption. (5) Besides clear added value in the context of diagnosis and treatment, AI should have a positive cost-benefit balance.

##### Opportunities

Assistance to radiologists was expected to add value by improving efficiency and accuracy in ongoing patient care and monitoring. Furthermore, added value was expected by the premise that AI could seamlessly merge multimodal data, contributing to disease detection, characterization of lesions, and monitoring treatment. This should add value, lead to lower health care costs, improve patient outcomes, or a combination of the aforementioned. Hospitals and health insurance companies benefit due to process optimization, resulting in lower costs and value-adding health care provision.

##### Frictions

With AI as a copilot assisting, and sometimes even taking over from the radiologist, the chance of discrepancies arises. On the one hand, participants indicated that AI is likely to make fewer mistakes than a radiologist and that AI should be trusted. On the other hand, in case a radiologist reaches a wrong conclusion with the suggestion of AI, the radiologist should not be allowed to hide behind the output of the AI, which creates a friction between efficient use of AI and increased responsibility in case of mistakes. As a solution, the participants suggested that the radiologist always retains the ultimate responsibility for the conclusion of the radiology report. This still requires checking the output, but was it considered faster than going through multiple series of images? For patients, it was envisioned that AI would lead to increased demand for data, which could lead to unwanted use of personal data. To prevent this, data users should receive explicit and flexible consent, meaning that data can be used only for the purpose and duration that a patient allows. Furthermore, introducing AI in radiology was expected to increase the detection of incidental findings, many of which may lack clear clinical significance. This raises complex ethical challenges, particularly when balancing a patient’s right not to know with the moral obligation to share potentially medically important findings. Participants in our study emphasized the importance of discussing the right not to know with the patients prior to imaging, as incidental findings can impose a psychological burden. They also stressed the necessity of keeping a human doctor in the loop, as the moral and clinical judgment required in these situations exceeded their expected capabilities of current AI systems and the demand for expertise.

### Scenario 2. Health Without Walls: AI and the Rise of (Mobile) Multimodal Screening

#### Materialization

The participants developed 2 separate ideas of extramural health checks: in one, they envisioned an environmentally sustainable, electric bus touring across the country, in a similar setup as the current population screening for breast cancer prevention in the Netherlands. Such a bus enables screening for a wide variety of potential diseases, which potentially benefits from large-scale screening programs. When AI detects a high-risk scan, the scan is forwarded to a radiologist, who decides whether to follow up. Within this scenario, it is possible to focus on the scan, or to combine the screening with blood or any other test, thereby combining data from multiple streams into a single risk assessment.

The other idea was focused on an extramural imaging, diagnosis, and treatment facility, which can, for instance, be located in a shopping mall. Citizens can visit the facility for a self-initiated check or after being invited due to specific risk factors, such as age. AI is subsequently used for fast diagnosis and forwards data to a radiologist if follow-up is required. As a consequence, there is still value in human intervention and responsibility. This scenario requires data to be shared with hospitals, for example, through electronic health records. A step further is to fully automate the diagnostic workflow by using large models that use large retrospective datasets and individual risk factors to screen for patients at risk and subsequently immediately treat correctly identified patients.

#### Moral and Economic Considerations

Participants emphasized how important they consider trust and responsibility when thinking about AI and radiology. Questions raised by a social scientist and patient representative included, “Is it responsible to leave decisions to computers?” (g1-s2) and “Who should be responsible for this?” (g1-s1). The ownership of the process and of the data was questioned. According to participants, AI can have added value in this scenario, including alleviating burdens from hospital staff, improving patients’ quality of life with an earlier start of the treatment, and equal access to health care.

Moreover, participants suggested that additional value can be calculated in added quality-adjusted life years, which can be translated into societal gains. The primary benefit of these scenarios may be that early diagnosis reduces long-term health care costs by shifting care from acute to preventive services. For funders, for example, the government, of large-scale AI-supported imaging-based screening programs, this shift may represent potentially favorable cost-benefit ratios where upfront investing may yield substantial savings and productivity over time. Additionally, commercial gain is possible, for example, for companies providing the bus with diagnostic hardware and software. Participants discussed various types of business models. Software companies responsible for evidence generation and validation can charge subscription- or use-based per license. Companies supplying scanners often operate on a purchase or lease model, and decentralized centers or scanner-on-tour suppliers could cover costs via direct-to-consumer supply or health insurance. For quality, competition between companies was regarded as crucial, and hospitals could consider purchasing an intramural diagnostic platform that supports multiple products from different vendors. This creates an overarching platform with competing products. Companies developing the software suites and the AI application that can be integrated into them should be prime movers here. Participants discussed the potential for the program to eventually become institutionalized, similar to breast cancer screening. Business models were seen as increasingly promising, especially once such programs began to generate large-scale data that can be leveraged to train data-hungry models.

if you have an AI algorithm that is CE-marked [Conformité Européenne], it doesn’t learn from the data it receives, right? That’s locked down. That’s what the CE marking is for. It only goes into development mode when you have data to further develop it on, and then, you do an update, and you put that into the system. So, it’s not like we always think that the AI just learns by itself.

[g1, s3]

#### Opportunities

Participants agreed that AI could offer an opportunity for the field that is in need of change, with the number of scans being requested rapidly increasing.

There is a report out right now that anticipates continuous growth in the years to come. Drivers are an aging population, more chronically ill patients, but also the increase in technological possibilities. At the same time, there is a growing demand coming from patients and the general population to have more insight into their health. Extramural health checks might be an answer to this demand.

[g1, s3]

Participants envisioned a future in which AI could more and better support the role of medical professionals. Decentralized health care facilities can play a large role in the health care system and help decrease the growth of private companies offering whole-body scans, which was deemed undesirable as disparities in income and wealth may translate into unequal access to health care services, potentially leading to disparities in health outcomes.

#### Frictions

Participants’ discussions shed light on a couple of frictions. First, they questioned whether the model should be organized in a centralized or decentralized manner, what the implications of decentralized testing would be for referring people to a hospital, and how data collected may or could be shared. Decentralized centers would need to build support within the broader network of health care institutions, hospitals, companies, and so on, for data sharing; they needed correct licensing, and interoperability must be addressed. Access and management of big data can create suspicions among patients, as they may think: “your data no longer belongs to you” [g1-s2].

Another potential friction arose around new role divisions and associated responsibilities. Laboratory technicians may outsource more tasks and perform fewer tasks themselves, radiologists may be hired on a consultancy (freelance) basis, and new roles may emerge (such as radiology assistants). This could increase the distance between the radiologist and the referring physician, which could complicate responsibilities and task distribution. A third friction point regarded sustainability:

What are we going to do? Is that desirable? So, I think we have to assess upfront, do you need a scan? Yes or no?

[g1-s3]

This question was answered later on:

AI can help with that as well. They can look in advance: from, does this patient need a scan? And then you are working more sustainably, because yes, the most sustainable scan is no scan.

[g1, s1]

On the one hand, there was a need to manage this rising demand, with patients starting to demand scans for more certainty

Because there are, of course, patients that ask for a scan, they [patients] want certainty.

[g1, s5]

versus the question of whether you need to better manage this demand. This can be done either by enlarging the role of AI and maximizing the use of the capacity of available scanners or by downsizing the demand by asking whether a scan is actually necessary, which is a decision AI can support in this scenario.

### Scenario 3. Early AD Insight: Proactive AI for a Longer Independent Life

#### Materialization

A group of participants chose to build their scenario around a concrete use case. The participants quickly decided to further develop their scenario around the idea that in the future, it becomes possible to predict, by analyzing either MRI data or only multimodal data, whether a citizen later in their life is likely to develop AD. This is achieved by screening citizens from a certain age group for the disease, either upon invitation or upon the citizens’ request (similar to the previous screening scenario). The benefit of using AI in very early stages to detect or predict AD, participants mentioned, can be that (future) patients are helped and trained to live independently or with minimal help for longer. This approach should improve the quality of life of patients, alleviate some of the societal burden of caregivers for these future patients, and may lead to reduced costs:

Allowing Alzheimer’s patients to function independently for as long as possible. [...] to keep support costs as low as possible.

[g3, s3]

Explainability was discussed as an important model aspect for patients. A patient representative argued that the model should be able to explain how it arrived at a certain decision. However, a participant with a background in pathology contextualized that this can be very task dependent. The researcher gave an example that was more related to her own field of expertise:

because for tumor detection, it [the AI] does not need to explain why something was flagged. ...Because we check it [the decision of the AI], so it is [in this case] AI-assisted. So I think: for this task, it does not need to explain what something is flagged for [...], because we check it [the flagged location].

[g3, s6]

The participants also briefly touched upon the business landscape, indicating that having multiple vendors would be a desirable aspect:

It’s better to have multiple suppliers. Otherwise, we will have a monopolist.

[g3, s3]

#### Moral and Economic Considerations

Participants found it important that implementing AI would be a meaningful addition to radiology, benefiting the individual patient. Thorough validation of the AI product can lead to improved trust in its use. Trust can also be enhanced through (human) explanation of the output. This means that either the algorithm should be able to explain its own output or radiologists should provide an explanation when a textual one is unavailable. Furthermore, patients should always remain in charge of their data and consecutive algorithmic outputs. That is, the data acquired upon clinical indication and the output of the algorithms should be shared only upon their voluntary consent. The participants also believed that individual patients or a society that shares personal data should monetarily benefit from commercial products. In other words, according to the participants, commercial parties should not be allowed to earn profits over products forever and profits should eventually flow back to society in some form, given that the AI product is essentially enabled by voluntarily provided personal data.

#### Opportunities

The participants argued that AI should deliver a product that radiology currently cannot. That is, true added value is achieved when AI detects what a radiologist cannot see, or when AI predicts future events that are not yet visible as pathology at the time of imaging. Participants discussed that the role of AI in this scenario is to assist with screening. Furthermore, predicting disease before it can manifest can lead to improved quality of life, specifically for AD. The participants highlighted that this is especially the case due to the human and emotional side of a progressive disease such as AD.

#### Frictions

The participants discussed the need for increased scanning capacity as a potentially negative consequence of AI. Granting all citizens the opportunity for screening for early detection of AD is costly, partly due to purchasing the scanners. However, using this additional scanning capacity can lead to improved quality of life for the patients, a lower societal burden for informal caregivers, and lower health care costs if cost-effectiveness of the screening is proven. The risk of incidental findings increases, though, which needs to be balanced with increased benefits of early detection. Whereas the industry employee argued that regulations can sometimes be experienced as burdensome, the patient representative found the regulations justifiable with respect to privacy, indicating that without strict regulations the patient representative may not share personal data at all.

Finally, the participants discussed potential consequences concerning insurance and autonomy: if AI predicts a future diagnosis such as AD, an insurance company may want access to those results, given that they may want to assess and price coverage accordingly. Also, the question was raised whether, “will they [some authority] say automatically that you move to an Alzheimer’s [care] house and you have to sell your home?” [g3, s5]. According to the participants, these aspects were undesirable, and these open questions warranted some serious thought.

### Emerging Themes

**Figure 1.**
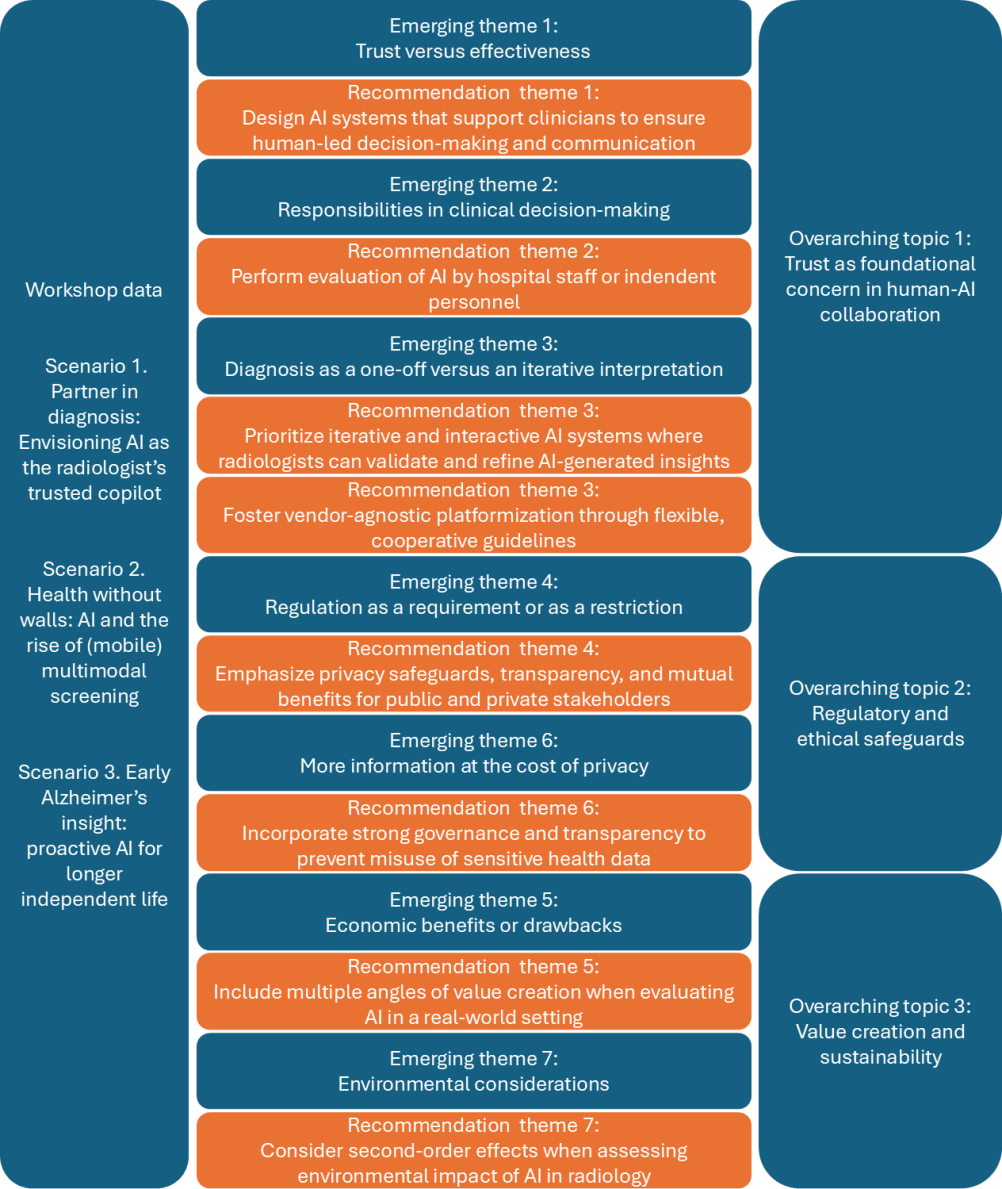
Summary of the workshop, 7 emerging themes, corresponding recommendations, and 3 overarching themes. AI: artificial intelligence.

Seven common themes emerged from the 3 future scenarios, along with their moral and economic considerations, opportunities, and frictions. Figure 1 summarizes the 7 emerging themes, recommendations based on the emerging themes, and 3 overarching themes.

#### Emerging Theme 1: Trust Versus Effectiveness

Seemingly, trust in AI represents a critical trade-off. On the one hand, trust could enable AI to live up to its potential as a stand-alone technology to effectively address complex tasks, such as medical diagnosis or large-scale hospital workflow optimization. On the other hand, patient representatives feared risk for automation bias. Additionally, participants discussed that excessive reliance on AI systems can diminish human supervision, increasing the clinician’s risk of automation bias and the erosion of critical decision-making capabilities. To break out of this trade-off and enhance trust, according to the participants, would often necessitate the adoption of interpretable and explainable AI systems, which radiologists can eventually check. Thus, achieving an optimal balance between trust and skepticism is essential to mitigate risks while harnessing the transformative potential of AI in radiology.

#### Emerging Theme 2: Responsibilities in Clinical Decision-Making

Implementing AI was considered a shared responsibility among stakeholders, each contributing distinct perspectives and expertise. Policy makers play a central role in establishing regulatory frameworks that guide legal-ethical boundaries for AI development and deployment. Industry players produce and scale practical AI applications within the established boundaries set by the aforementioned policy makers. However, when it comes to clinical decision-making, the participants foresee that medical specialists and health care workers will ultimately be responsible for the health and safety of their patients, and clinicians also feel that way. To reach this goal, it could be beneficial for medical specialists to cooperate with more technically oriented hospital personnel to perform quality assurance tests, particularly to ascertain that AI performs equally well on local datasets compared with how industry markets the performance of their products. Furthermore, according to the participants, there might be a conflict of interest when industry performs its own quality assurance. Thus, local testing may be a critical requirement for safe deployment and responsible use of AI in radiology and, according to the workshop participants, should be performed by independent hospital staff.

#### Emerging Theme 3: Diagnosis as a One-Off Versus an Iterative Interpretation

Given the rapid development of AI, radiological diagnosis is increasingly shaped by algorithmic output. These outputs are often treated as static and definitive, similar to finalized radiology reports. The static nature of AI’s output presents 2 interrelated shortcomings. Radiological diagnosis is often not a fixed product but a dynamic process. It relies on expert interpretation and is influenced by evolving clinical information, such as neurological examinations and pathology results. Several participants noted that diagnosis often emerges through iterative reasoning and collaboration with other medical specialists. AI systems, which typically generate preanalyzed findings or diagnostic probabilities, risk oversimplifying this complexity. Participants suggested that integrating multimodal data could help AI better reflect current clinical practice. In addition, static outputs could lead to a greater demand for diagnostic certainty, potentially leading to unnecessary follow-up: by detecting more abnormalities and including findings that may never become clinically relevant, AI could lead to more scans, consultations, and interventions. A potential solution to both shortcomings that was suggested by participants is a “human-in-the-loop approach,” where the radiologist remains responsible for interpreting AI outputs within the broader clinical context. Collaborative frameworks in which AI complements rather than replaces human expertise were seen as essential. These should include clear protocols for follow-up, shared decision-making with patients, and the development of transparent and explainable AI systems to support trust and appropriate clinical action.

#### Emerging Theme 4: Regulation as a Requirement or as a Restriction

Regulations play a dual role in technological innovation, acting both as a potential constraint and as a necessary enabler for progress, particularly in a fast-paced field such as AI in radiology. On the one hand, industry employees indicated that overly rigid or premature regulations can restrict innovation by imposing burdensome compliance requirements. On the other hand, patient representatives argued that well-designed regulatory frameworks are essential to ensure safety, fairness, and public trust—key requirements for adopting novel technologies. For example, as indicated by patient representatives, robust protection of patient privacy not only follows an ethical imperative but also creates a secure environment for patients to share their data. In other words, effective regulations are required for patients to feel safe when consenting to sharing their personal data, which is the cornerstone for all AI developments. Adaptive and dynamic regulatory approaches, capable of evolving alongside technological advances, were thus considered crucial for maintaining this balance. Following our workshop results, participants regarded effective regulation as an enabler rather than an obstacle to innovation, because it provides the foundation for trust necessary for groundbreaking technologies to thrive responsibly.

#### Emerging Theme 5: Economic Benefits or Drawbacks

The integration of AI in radiology presents significant economic opportunities and challenges. Following initial investments in hardware infrastructures and software packages, there is a wide variety of potential applications on a hospital level, societal level, and vendor level that could yield a net economic benefit. Participants emphasized that AI could significantly improve operational efficiency within hospitals. By automating routine tasks, such as appointment scheduling and administrative workflows, AI has the potential to reduce labor costs and streamline internal processes. These improvements may lead to better resource allocation and increased throughput, contributing to overall cost-effectiveness in clinical operations. At the societal level, the economic benefits of AI in radiology are diverse and potentially far-reaching. Participants pointed to several ways in which AI could contribute to more efficient health care delivery and improved population health. For example, timely diagnosis may enable earlier initiation of therapy, which can improve outcomes and reduce the need for prolonged or intensive treatment. Similarly, more personalized care, which could be supported by AI’s ability to integrate and analyze complex data, may help avoid unnecessary interventions and enhance the overall effectiveness of health care services. These developments could contribute to long-term cost savings and a more sustainable use of health care resources. However, participants also cautioned that increased diagnostic sensitivity might lead to more incidental findings, which could raise demand for follow-up care and specialist input, potentially increasing overall health care expenditures. According to some participants, the responsibility for initial economic value assessments of AI systems lies with the vendors, who should estimate whether their solutions offer sufficient value before deployment. However, participants emphasized that independent researchers should conduct postdeployment evaluations. Similar to quality assurance processes, this separation is essential to avoid conflicts of interest and ensure unbiased assessments of economic performance.

#### Emerging Theme 6: More Information at the Cost of Privacy

Unintended consequences from AI might arise when the decision becomes binding rather than advisory. This decision could drastically affect a patient’s life. In the AD case, the participants discussed the extreme example of mandatory compliance with the AI decision for future patients when AD is predicted. Furthermore, if (by obligation) shared with insurers, these screening programs could have negative consequences for monthly health insurance premiums, thus penalizing individual citizens financially for factors beyond their control, which raises privacy, fairness, and proportionality concerns. Or, in the extreme case of extrapolating this issue, insurance companies may increase costs for patients at risk or deny (future) patients access to (additional) health and property insurances at all. Regulation should therefore be in place to prevent such a scenario from materializing. Moreover, patients have the right to not know. The participants agreed that this moral dilemma might be quite difficult for an AI to weigh, where there needs to be a good balance between medical necessity and patients’ rights and preferences. To counter this unintended consequence, human intervention might be the only solution.

#### Emerging Theme 7: Environmental Considerations

Finally, it was discussed that AI is an energy-demanding technology. Combining AI with radiology for screening means that scarce resources might be used for citizens who may not (yet) express any disease symptoms. Thus, the participants discussed potential benefits for health care and the economy of AI in radiology should also be carefully weighed against the potential environmental impact. The monetary gain and gain in quality of life after the introduction of AI to radiology should therefore be proportionate to the environmental impact of additional interventions, such as screening in combination with AI.

## Discussion

### Principal Findings

In this manuscript, we report and reflect on a qualitative cocreation workshop we designed and conducted, inspired by responsible innovation approach, aiming to explore future scenarios for AI in radiology, their opportunities and frictions, by involving a variety of stakeholders in the field of radiology. Interestingly, in imagining futures of AI in radiology, the majority of the groups, in one way or another, came up with a scenario to upscale radiology via screening or extramural imaging to early detect and prevent pathology from becoming a disease. However, there were differences in the imagined role of these applications, and of actors in the field, including governments, insurers, technology developers, and citizens. By including a wide variety of topics, we aimed to broaden the participants’ scope of thinking. Resultingly, our participants discussed moral and economic considerations, envisioned opportunities, and foresaw frictions arising related to interconnected topics on trust, responsibilities, workflows, regulatory requirements, dealing with economic benefits, and unintended consequences.

### Comparison With Prior Work

#### Trust Versus Effectiveness

In our analysis, we observed trust in AI as a complex topic the participants linked to, for example, performance and explainability. The participants perceived a paradoxical relationship between trust and effectiveness: the more trust they were willing to place in AI, the more AI could potentially operate as a stand-alone technology in radiology and fulfill its promise of improving clinical outcomes. Yet, this very trust introduces ethical tensions. As described by Singh et al [77], the ethical paradox in medical practices arises when efforts to enhance health care through AI risk diminishing the humanistic aspect of care, such as empathy and interpersonal interactions, thereby potentially provoking skepticism and decreasing trust. Reflecting on scenarios 1 and 2, AI could be deployed for triaging or identifying patients at risk. As such, AI applications may allow clinicians to focus their limited time on more complex and emotionally sensitive cases, potentially improving overall effectiveness of care and patient well-being, which were expectations that were reported earlier as well [37]. However, fully trusting AI may lead to dependency on AI or potential loss of human oversight. Similar concerns were also shared during the cocreation workshop. Patient representatives, among other stakeholders, confirmed that a human-in-the-loop was, in their opinion, still required. This idea was also shared in earlier research [63,78-80], although in other reports, patients have also indicated that autonomous AI could be acceptable on the condition that AI outperforms a radiologist [18]. Yet, patient representatives and other stakeholders seemed to have a positive attitude toward AI-assisted triaging, especially because the ultimate decision remains with a radiologist. The explainability of the AI-based results of the triaging could further help gain clinicians' confidence [53]. Discussions suggest that following these conditions, applications of AI in radiology may contribute to enhancing efficiency without compromising safety or trust. Based on the links between effectiveness, trust, explainability, and the humanistic aspect of care, we recommend designing AI systems that support clinicians, ensuring human-led decision-making and communication, while leveraging AI for effectiveness.

#### Responsibilities in Clinical Decision-Making

Participants in the workshop highlighted that there should not be conflicts of interest in the case of responsibilities. In other words, in environments where people must act responsibly to, for example, safeguard patients’ health, one should not be incentivized to act differently. For this reason, some participants emphasized that generating evidence for AI in early stages of development and real-world testing of AI should be performed by 2 distinct parties. Whereas industry was seen as responsible for product (co)development and early evidence generation, real-world implementation, thorough (local) testing, and longitudinal evaluation should be performed by independent hospital personnel, potentially in collaboration with more technically oriented personnel such as clinical physicists or technical physicians [81]. Furthermore, there was a seeming distrust of commercial parties to perform their own prospective evaluation. Earlier research focused on *how* to perform postinstallation evaluation [22,26,82-84]. However, this research did not dive into *who* should do so and what the possible impact on interconnected themes such as trust could be. Specifically, industry involvement should be limited in prospective evaluation, such that thorough evaluation can be performed with as little conflict of interest as possible. Our research demonstrates preliminary evidence that independent evaluation of AI in a clinical setting is pivotal to enhancing trust, and that trust can be achieved only by proper distribution of responsibilities along the innovation, installation, and evaluation chain. Therefore, we recommend that evaluation of AI is performed by hospital staff or independent external parties.

#### Diagnosis as a One-Off Versus an Iterative Interpretation

The proposed function of the AI within the radiology system varied within our workshop, ranging from screening to acting as a copilot for the radiologist. AI tools in medical imaging have traditionally functioned as narrow, outcome-focused instruments, for example, detecting polyps, segmenting tumors, or flagging anomalies in isolation [85]. However, radiology is fundamentally a process of evolving clinical interpretation, where patients’ histories, laboratory results, physical examinations, and ongoing changes in condition contextualize each series of images [86,87]. Multimodal data integration could enable AI to move beyond static predictions and could facilitate evolving model outputs [88]. Furthermore, the inclusion of large language models (LLMs) could provide narrative understanding, enabling AI to synthesize diverse inputs into coherent clinical insights [89-91]. In turn, a radiologist can check the output of the LLM or interact with the LLM, which could lead to further explainability and could foster trust, which also links back to emerging theme 1. This convergence transforms AI from a tool that offers answers into one that participates in reasoning. Importantly, this shift could allow AI to align more closely with the clinician’s cognitive workflow, adapting dynamically as new data come in [89-91]. It could also open the door to more explainable and human-centered AI systems that support, not replace, clinical judgment, as was also proposed earlier [63,78-80]. Thus, we recommend prioritizing iterative and interactive AI systems where radiologists can validate and refine AI-generated insights, ensuring that AI acts as a reasoning partner rather than a black box tool.

Moreover, the participants deemed the integrability of AI tools within existing hospital systems as a facilitator for successful adoption, as was also reported in earlier research [59]. Integrating these capabilities into unified platforms, rather than isolated tools, could thus further aid in the commercialization of AI in radiology. This process of platformization supports scalability, continuous learning, and interoperability [69], potentially enabling a shift from a one-off solution to comprehensive decision support ecosystems that are clinically and economically sustainable. Finally, participants regarded having multiple vendors as beneficial to the health care system, encouraging competition in either performance or costs, potentially benefiting patients. To support this, platforms should remain vendor-neutral, allowing easy integration from multiple providers. Whereas earlier research recommends four components: (1) (automatic) system triggering, (2) retrieval of relevant imaging series, (3) application of algorithm pipeline, and (4) availability of the results in Picture Archiving and Communication System of dedicated viewing software [92], we recommend conducting more research in the direction of cooperative guidelines that allow vendors to provide innovative solutions while remaining flexible for competition, potentially including regulatory sandbox environments, adaptive regulations, and value-based procurement.

#### Regulation as a Requirement or as a Restriction

In some discussions, the role of regulations came forward. While industry employees regarded regulations as a potentially impeding innovation, patient representatives regarded regulations as a requirement to share data. This paradoxical friction was also described in a recently published paper. According to Singh et al [77], the solution seems to be to strike the right balance between data accessibility and the obligation to safeguard patient confidentiality and to adhere to regulatory frameworks. This finding, previously reported among medical practitioners, was also identified in patient representatives, further confirming and reinforcing its broader relevance. The data paradox is quite complex: on the one hand, patient representatives in our workshop mostly preferred to retain ownership of their data. On the other hand, industry requires a diverse and up-to-date dataset, such that the AI products they develop generalize sufficiently well on various populations and do not suffer from data drift [40,46,51]. In light of this data paradox, future work should explore how hospital-private sector collaborations can be structured to enable responsible data sharing, with strong privacy safeguards, transparency, and mutual benefit for both public and private stakeholders. Recent global frameworks, such as the World Health Organization’s regulatory pillars for AI in health [93], the EU AI Act’s classification of medical imaging AI as “high-risk” [94], and international consensus initiatives such as FUTURE-AI [26], emphasize that striking this balance is not only desirable but also essential for compliance and trustworthiness. These frameworks advocate transparency, human oversight, and robust governance, which can guide collaborations toward ethical and legally sound data sharing.

#### Economic Benefits or Drawbacks

During the workshop, participants raised the question of whether it was desirable for companies to earn money using voluntarily provided individual patient data without patient populations benefiting. A competitive landscape was described by the participants that could solve these issues. First of all, multiple vendors compete for the best product. The participants argued that this aspect should lead to better health outcomes for individual patients, linking to earlier argued ethical justification of the use of patient data if companies contribute to public health benefits [95]. In other words, the profits earned using patient data are reinvested in the companies to improve existing products or to create new products, which benefit both public and private stakeholders. Second, vendors can compete for the lowest price, which benefits the health care system as a whole, as price competition can lead to lower health care costs. At the same time, a diverse vendor landscape may help mitigate risks associated with vendor lock-in [96], ensuring that health care providers retain flexibility in choosing and switching between solutions as technology evolves and needs change. However, without more studies rigorously evaluating AI systems in real-world clinical settings, it is difficult to substantiate claims of added value [74]. We therefore advocate for more studies that evaluate AI in a real-world setting and that include multiple angles of value creation, such as decreased reporting times, added number of relevant incidental findings, and the prevention of unnecessary follow-up scans or interventions. This should lead to elaborated business models for both vendors (suppliers) and hospitals (buyers). Upon positive results, these studies could further facilitate AI adoption in clinics and provide vendors with useful insights about the possibility to generate revenue with their products. One example comes from Brix et al [11], who found that AI-based image reconstruction could lead to improved patient throughput, thereby obviating the need for an additional scanner. Such insights can help vendors to demonstrate the added value of their AI solutions, which may justify premium pricing and unlock new business models, especially when validated in real-world clinical environments.

### More Information at the Cost of Privacy

Participants worried about data privacy, especially over the sharing of diagnostic and prognostic information inferred from AI with insurance companies, hinting that insurance companies might misuse this information. A recent review by Botha et al [97] also endeavored to examine the implication of AI tools on insurance. Whereas many aspects concerning insurance coverage were discussed, including the potential rise in insurance costs with the introduction of AI in clinical settings, second-order effects such as sharing information of future disease with insurance companies were underrepresented [97]. A perspective paper from 2018 by Char et al [60] warned of this looming issue, and our results demonstrate that these worries are still unresolved. Thus, a deeper dive into this specific topic seems warranted. Emerging literature confirms these concerns, highlighting risks of insurance-related discrimination from predictive analytics [98]. Regulatory frameworks such as World Health Organization’s guidance [93] and the EU AI Act [94] stress the need for strong governance and transparency to prevent misuse of sensitive health data. Incorporating these principles into practice could help mitigate ethical risks while enabling responsible innovation.

### Environmental Considerations

Another worry that came forward was the energy consumption of AI, which according to the participants potentially makes it an environmentally unsustainable solution. Whereas it is likely a correct assumption that adding AI to the radiology workflow increases energy consumption compared with a workflow without AI, efforts to make both algorithms and hardware more energy-efficient might help reduce the energy consumption for each inference [99]. Furthermore, AI could aid in reducing environmentally harmful gadolinium-based contrast agent use [100], or can reconstruct images from accelerated (and thus less time-consuming and less energy-consuming) MRI acquisitions [11,101], which both potentially have a net positive effect on the environment, as it may result in fewer MRI systems in use and less energy consumption per scan. Thus, whereas the participants’ worries are justifiable, these second-order effects also need to be accounted for when assessing environmental impact for AI in radiology.

### Limitations and Future Directions

The main limitation of this study is that we draw on a singular empirical case study set in a Dutch tertiary care center. Future studies should revisit our research questions in different countries and look at other care settings, including trans- and extramural settings. These other care centers are of special importance, since multiple participants independently hinted at the potential for extramural imaging facilities. Also, no insurance company employees could attend the workshop, despite efforts to include at least one. Insurance companies are a key stakeholder in the field, so their perspectives are potentially valuable in discussions on the demand side (reimbursement and assessment of added therapeutic value). Next, in future research, we recommend including patient representatives again, as their insights were invaluable to this work. Finally, by checking the audio recordings in full by 2 of the authors (MBS and SH) and refining transcripts and translations, we aimed to minimize potential nuance loss. Although some loss of data could still have occurred, these steps helped preserve the richness of the participants’ contributions as much as possible.

### Conclusions

With this paper, we aimed to explore wider stakeholder perspectives on futures of AI in radiology and to discuss the moral and economic considerations, opportunities, and frictions. The interaction between participants resulted in scenarios about an AI copilot, AI-enhanced extramural scanning, and AI-based AD screening. It prompted debates on perspectives that may not have emerged from isolated stakeholder analyses. Seven themes emerged from the analysis: (1) trust and efficiency of AI technologies, (2) responsibilities in clinical decision-making when AI is involved, (3) diagnosis as a one-off versus an iterative process, (4) regulations as a requirement or a restriction, (5) economic benefits or drawbacks, (6) trade-off between amount of information required and patient privacy, and (7) environmental considerations. In reflecting on the 7 emerging themes from the multistakeholder cocreation workshop, 3 overarching topics can be identified that help reflect on the broader implications of AI in radiology.

First, trust emerged as a foundational concern in human-AI collaboration. Participants emphasized that in their opinion, AI should support, not replace, clinical decision-making. Trust was seen as conditional for AI to be applied in radiology efficiently, meaning that explainability and the continued presence of a human-in-the-loop are deemed important. This human-machine model aligns with radiological diagnoses of iterative nature and was considered essential for safe and ethical implementation. Second, regulatory and ethical safeguards were central to discussions on data use, responsibility, and privacy. Stakeholders expressed diverging views: while industry employees saw regulation as a potential restriction, patient representatives viewed regulations as a requirement for data sharing. The friction between innovation and regulation can be dealt with through adaptive regulatory frameworks that evolve alongside technological capabilities while safeguarding patient autonomy and algorithmic fairness. A third topic relates to value creation and sustainability. Participants discussed the potential for AI to improve efficiency, reduce costs, and enhance diagnostic accuracy. However, concerns were raised about incidental findings, data monetization, viability of business models, and the energy demand of AI systems. Platformization and vendor-neutral integration were seen as a way to foster innovation while maintaining affordability and scalability. Moreover, participants advocated for competitive ecosystems where multiple vendors could contribute to better outcomes and lower health care costs.

Together, these overarching topics offer a lens through which future research and policy can address the complex interplay of technological potential, ethical responsibility, and systemic sustainability in AI-driven radiology. Future research is needed to investigate the generalizability of our findings in different countries and settings, or to address open questions on real-world economic benefit and sustainability.

## Supplementary material

10.2196/83407 Multimedia Appendix 1 Worksheet canvas for exercise 2. The original worksheet during the workshop was in Dutch.

10.2196/83407 Multimedia Appendix 2 Worksheet canvas for exercise 3. Mind that, again, the original worksheet during the workshop was in Dutch.

10.2196/83407 Multimedia Appendix 3 Quotebook.[Aff aff1]

## Abbreviations

AD: Alzheimer disease
AI: artificial intelligence
LLM: large language model
MRI: magnetic resonance imaging
