# Supplementary figures and images for: Anticipating Moral and Economic Considerations, Opportunities, and Potential Frictions for AI in Medical Imaging: Multistakeholder Cocreation Study
Source: J Med Internet Res. 2026 Feb 25;28:e83407. doi: 10.2196/83407 (PMC12935424; doi:10.2196/83407)

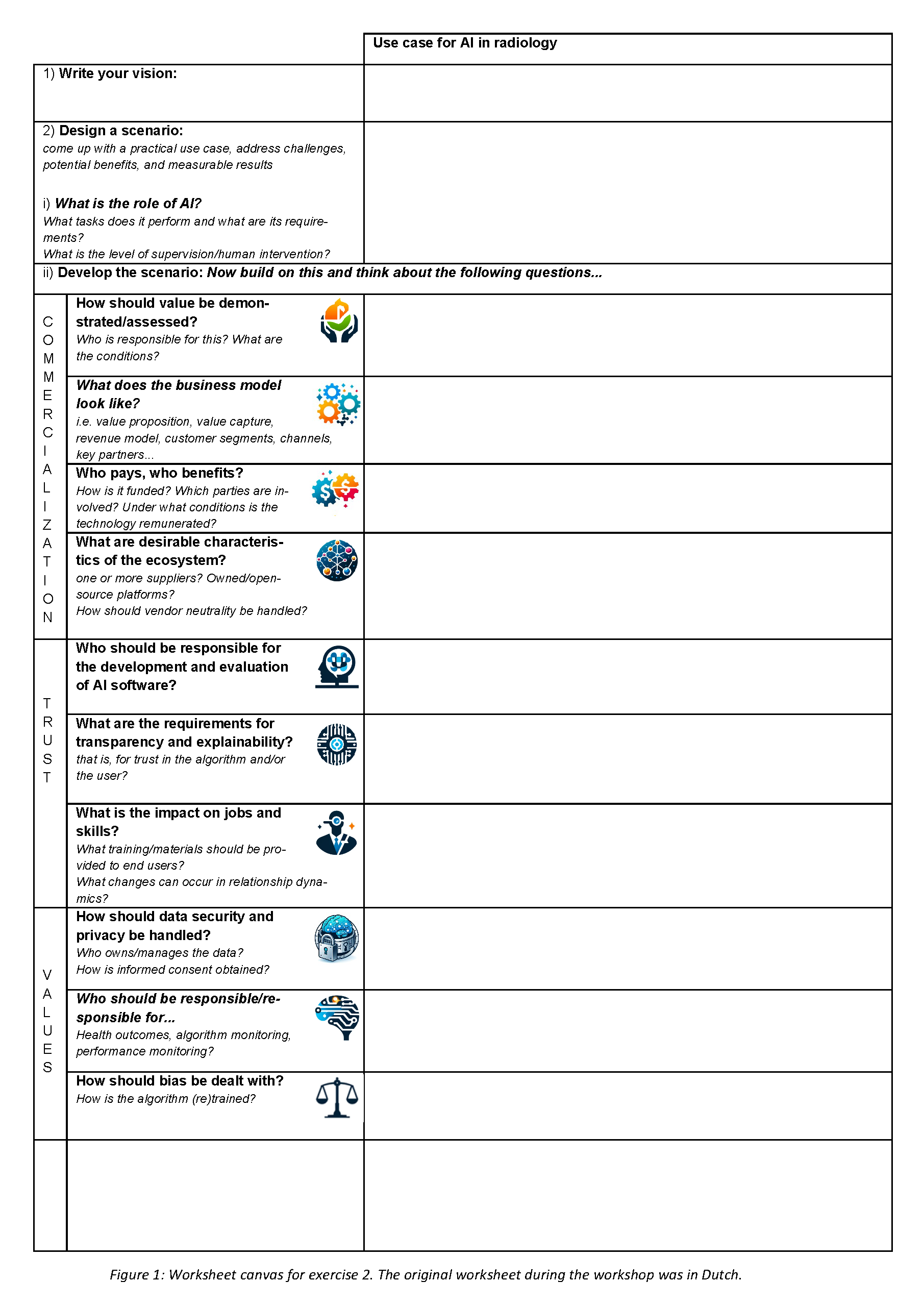

Supplement: Multimedia Appendix 1 [file jmir-v28-e83407-s001.png]

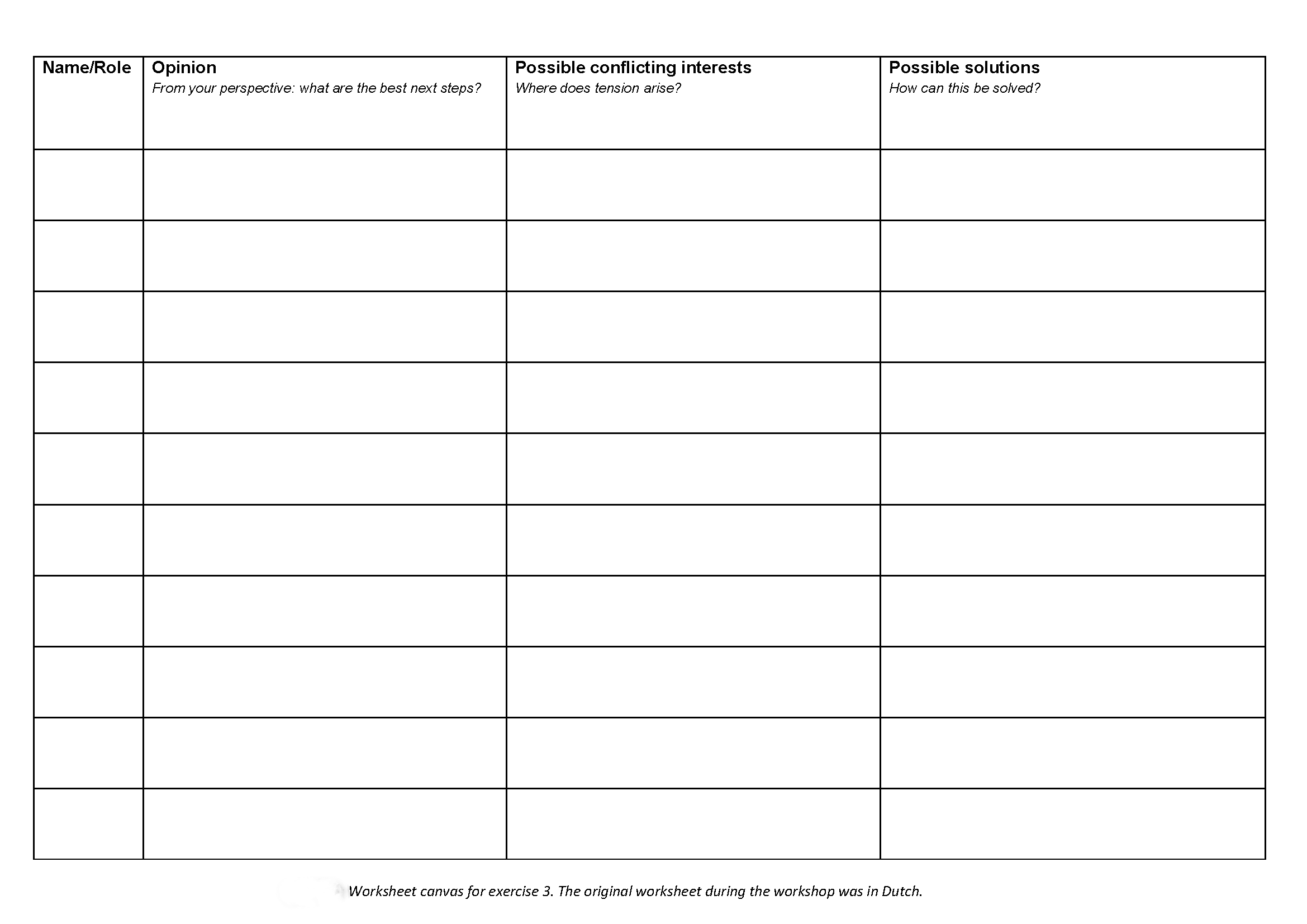

Supplement: Multimedia Appendix 2 [file jmir-v28-e83407-s002.png]
