# Supplementary material for: Anticipating Moral and Economic Considerations, Opportunities, and Potential Frictions for AI in Medical Imaging: Multistakeholder Cocreation Study
Source: J Med Internet Res. 2026 Feb 25;28:e83407. doi: 10.2196/83407 (PMC12935424; doi:10.2196/83407)
Supplement: Multimedia Appendix 3 [file jmir-v28-e83407-s003.docx]

# Multimedia Appendix 3. Exemplar quotes per emergent theme.

## Emerging theme 1: Trust versus effectiveness

Quote 1: “because for tumor detection, it [the AI] does not need to explain why something was flagged. … Because we check it [the decision of the AI], so it is [in this case] AI-assisted. So I think: for this task, it does not need to explain what something is flagged for […], because we check it [the flagged location]” (g3, s6).

Quote 2: [in a discussion on triaging scans]: “In case of high risk [detected by AI], the scan can be forwarded [to the radiologist]” (g1, s4)

Quote 3: “And maybe I do not even have to look at it. It [the AI] automatically judges change, and you get [to see] a curve. This [lesion] is larger, that [lesion] is smaller. And in the end I check if I agree.” (g2-s3)

Quote 4: “It depends on how trustworthy the AI is. If it is very trustworthy, the human only needs to agree to use it [the output]. Human oversight in the clinic may diminish to almost zero.” (g2-s3)

## Emerging theme 2: Responsibility in clinical decision-making

Quote 5: “Is it responsible to leave decisions to computers?” (g1-s2)

Quote 6: “We [industry] perform our own studies for our products to clinically validate them. We have to submit them to the FDA for the United States of America and at the EMA for Europe” (g2-s1)

## Emerging theme 3: Diagnosis as a one-off versus an iterative interpretation

Quote 7: “…also the integration with biomarkers, with pathology with those [foundational models], and the EHR [Electronic Health Record], gives a much more complete picture than just with those images.” the workshop participant continued, arguing how that despite all clinicians already work together in the same system, she expects AI to be an enabler of actual integration : “(…) I think that integration into AI of all those different things will also become very strong“ (g3, s6).

Quote 8: “That you do not only look at radiology, but also at pathology, or even at all available data of a patient. That all this data is included. Because radiology only seems vague, where we make decisions based on images. We could use more sources of information. The specialism radio-pathologist does not exist, but I believe that we could integrate that with AI.” (g2-s3)

Quote 9: “My vision is faster and better use of AI. And simplified use of all AI applications that are now invented. It’s quite logical that diagnostics, characterization and detection of change, where you have scan, and AI tells me: ‘Here I found an anomaly, I believe this is the diagnosis, what do you think?’ But if I then want to change the diagnosis, the process will change.” (g1, s4)

## Emerging theme 4: Regulations as a requirement or as a restriction

Quote 10: “your data no longer belongs to you” (g1-s2).

Quote 11: “if you have an AI algorithm that is CE-marked [Conformité Européenne], it doesn’t learn from the data it receives, right? That’s locked down. That’s what the CE marking is for. It only goes into development mode when you have data to further develop it on, and then, you do an update, and you put that into the system. So, it’s not like we always think that the AI just learns by itself” (g1-s3).

Quote 12: “If it were up to me I would like to have my data anonymized for scientific research. That to me is a necessity.” (g3-s2)

## Emerging theme 5: Economic benefits or drawbacks

Quote 13: “It's better to have multiple suppliers. Otherwise, we will have a monopolist.” (g3, s3)

Quote 14: “Allowing Alzheimer's patients to function independently for as long as possible. […] to keep support costs as low as possible” (g3, s3).

Quote 15: “I think for screening, the government will profit if all becomes more efficient. And then the whole Netherlands profits” (g1, s7) “The government pays, be could also earn this [investment] back. More QUALYs [quality adjusted life years], healthier workforce, but also economic growth. But if the government pays, some other party will earn, which is the provider of AI” (g1, s4)

## Emerging theme 6: More information at the cost of privacy

Quote 16: “will they [some authority] say automatically that you move to an Alzheimer’s [care] house and you have to sell your home?” (g3, s5).

Quote 17: “How far can we go in anonymization? For instance, with MRI scans, you would be able to reconstruct a face [of a patient].” (g1, s7)

Quote 18: [on a discussion on government-funded AI-enhanced screening and algorithm ownership] “Well, maybe the government thinks that they should be owner of the AI algorithm, and maybe they could benefit from all data that is acquired during this screening, as this data could be used to further improve the algorithm, such that they can work towards a super-algorithm for screening.”

## Emerging theme 7: Environmental considerations

Quote 19: “What are we going to do? Is that desirable? So, I think we have to assess upfront, do you need a scan? Yes or no?” (g1-s3).

Quote 20: “AI can help with that as well. They can look in advance: from, does this patient need a scan? And then you are working more sustainably, because yes, the most sustainable scan is no scan” (g1-s1).

Quote 21: [From a discussion on the use of electric busses for population screening.] “There is less travel to location [hospitals], because the scan comes to you. This is an argument in favor of more sustainability [when using a bus instead of individual travel]” (g1, s 2). “That is true, however, we also generate a lot of data and storing this data costs a lot of energy (g1, s4).”
